# Comparison of angiographic outcomes and complication rates of WEB embolization and coiling for treatment of unruptured basilar tip aneurysms

**DOI:** 10.1038/s41598-022-15113-w

**Published:** 2022-06-28

**Authors:** Erkan Celik, Yigit Ozpeynirci, Thomas Liebig, Marc Schlamann, Franziska Dorn, Nils Lehnen, Eberhard Siebert, Lukas Goertz, Christoph Kabbasch

**Affiliations:** 1 Department of Radiology and Neuroradiology, University Hospital of Cologne, Cologne, Germany; 2 Department of Neuroradiology, Ludwig’s Maximilian University Munich, Munich, Germany; 3 Department of Neuroradiology, University Hospital Bonn, Bonn, Germany; 4 Department of Neuroradiology, University Hospital of Berlin (Charité), Berlin, Germany

**Keywords:** Neurovascular disorders, Characterization and analytical techniques

## Abstract

Endovascular coiling represents the standard treatment for basilar tip aneurysms. Some of these aneurysms are not amenable to conventional coiling due to a complex aneurysm geometry, hence, novel devices such as the Woven Endobridge (WEB) have been developed. We retrospectively compared WEB embolization and coiling for the treatment of unruptured basilar tip aneurysms. Patients treated with WEB or coiling at four centers were reviewed. Procedure-related complications, clinical outcome and angiographic results were retrospectively evaluated and compared. Forty patients treated with the WEB and 35 patients treated by coiling were included. Stent-assistance was more often necessary for coiling than for WEB embolization (71% vs 2.5%, p < 0.001). The technical success rates were 100% for both methods. The overall complication rates were not significantly different between groups (WEB: 5%, coil: 11%, p = 0.409). Procedural morbidity rates were 9% in the coiling group and 2.5% in the WEB group (p = 0.334). There was no mortality. Treatment duration was shorter for WEB implantation than for coiling (p = 0.048). At mid-term follow-up, complete occlusion, neck remnants and aneurysm remnants were observed in 89%, 4% and 7% for the WEB, respectively, and in 100%, 0% and 0% for coiling. While complication rates and mid-term angiographic outcome was comparable between the groups, the WEB was associated with a shorter treatment duration and required stent-assistance less frequently. The choice of the treatment modality should be made based on the specific aneurysm characteristics, the individual experience of the neurointerventionalist and patient preference.

## Introduction

Basilar tip aneurysms (BTA) account for approximately 8% of all intracranial aneurysms^1,2^. BTA aneurysm rupture results in subarachnoid haemorrhage (SAH) and leads to a mortality rate up to 23%^3,4^. Due to their proximity to the brainstem and the narrow surgical corridor, the treatment of BTA by microsurgical clipping has become obsolete and endovascular therapy has evolved as the treatment of choice.

Conventional coiling is often not feasible in BTAs due to an unfavourable dome-to-neck ratio. Moreover, in aneurysms with suitable geometry, sole coiling may be associated with recanalization rates up to 40%^5–8^. In more complex aneurysms, additional stent implantation can prevent coil protrusion into the parent artery and may allow for a denser coil packing^9,10^. However, stent implantation can be technically complex at the basilar tip because of the usually broad-necked configuration and incorporation of more than one vessel origins into the aneurysm. Remodelling of the basilar tip is necessary in these cases and can be achieved by modification as Y- or T-stenting^11^. Compared to stand alone-coiling, SAC has proven higher aneurysm obliteration rates, however complication and morbidity are described to be slightly higher in SAC^12^. A further drawback of stent-assisted coiling is its need for permanent anti-aggregate therapy^13^.

The Woven Endobridge (WEB; Sequent Medical, Aliso Viejo, CA, USA) is a barrel-like self-expandable device made of nitinol wires that has been developed for treatment of broad-based bifurcation aneurysms. Within the aneurysm sac, the WEB provides immediate flow-disruption through the aneurysm ostium, which typically results in subsequent aneurysm thrombosis and neo-endothelialization in the neck region over time^14^. As the WEB position is completely intrasaccular, anti-platelets are not necessarily required after treatment. Several studies demonstrated a reasonable safety and efficacy profile of the WEB for both ruptured and unruptured intracranial aneurysms^15–20^.

The objective of this study was to compare the WEB with conventional coiling with or without stent-assistance for the treatment of unruptured BAT aneurysms in terms of procedural complications, clinical outcome and mid-term angiographic results. Only aneurysms that were amenable by either treatment modality were included.

## Methods

This is a retrospective study of consecutive patients treated for unruptured basilar tip aneurysms with the WEB or coiling at four German high-volume neurovascular centers (January 2011–March 2021). The study protocol was approved by the local ethics committee of the University Hospital of Cologne. The need for informed consent was waived by the local ethics committee of the University Hospital of Cologne. The study was conducted in accordance with the STROBE guidelines in compliance with the national legislation and the Code of Ethical Principles for Medical Research Involving Human Subjects of the World Medical Association (Declaration of Helsinki).

### Inclusion and exclusion criteria

**Figure 1:**
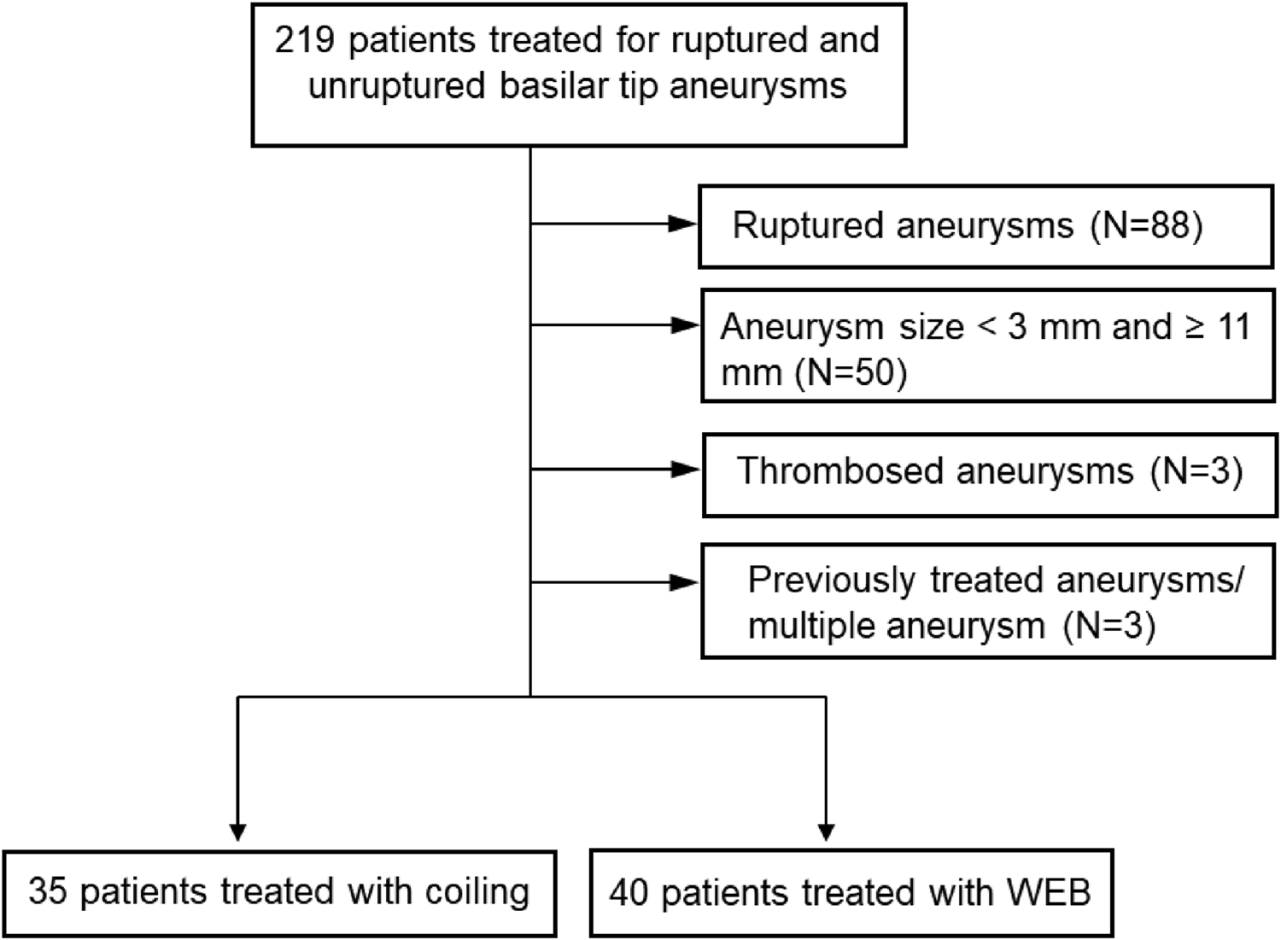
Patient’s selection flow chart.

All unruptured basilar tip aneurysms treated by WEB or coiling with or without stent- or balloon assistance were retrospectively identified. These aneurysms were reviewed by a blinded neurointerventionalist (C.K.) to identify aneurysms that were amenable to both WEB embolization and coiling. Only these aneurysms were included. Exclusion criteria were: (1) aneurysm size < 3 mm and ≥ 11 mm, (2) partially thrombosed aneurysms, (3) multiple aneurysms treated during one session, and (4) previously treated aneurysms (Fig. 1).

### Procedure

At each center, the treatment indications were based on a discussion with the patient and an interdisciplinary neurovascular board decision, consisting of neurologists, neurointerventionalists and neurosurgeons. The decision to treat aneurysms with the WEB was left to the neurointerventionalist’s discretion and mainly dependent on the width of the aneurysmal base. Informed consent for the procedure was obtained from all patients. Coiling was predominantly employed in the first half of the study period, whereas WEB embolization was predominantly used in the second half.

**Figure 2:**
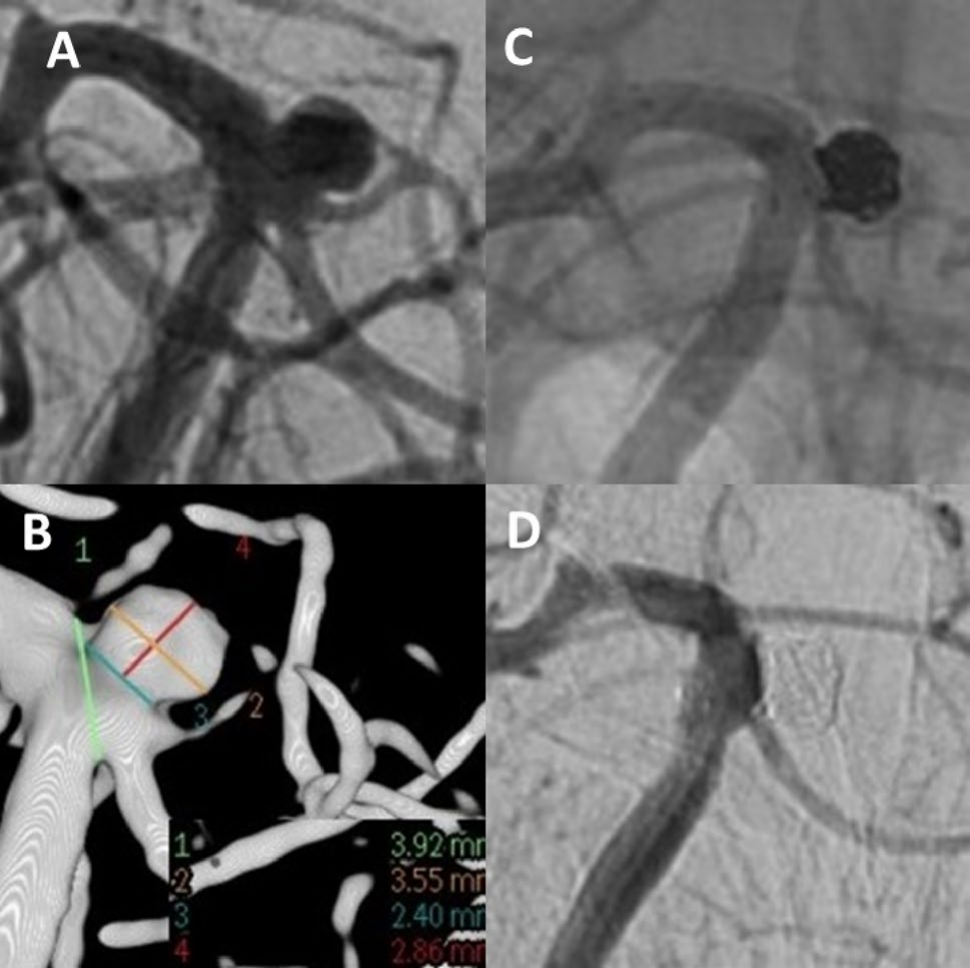
Unruptured aneurysm at the basilar tip (**A + B**). Stent-assisted coiling was intended. A stent (barrel-3550) with a barrel-shaped central segment was inserted to bridge the aneurysm neck in such a way that both the aneurysm base and the doubled superior cerebellar artery on the left are secured. The aneurysm sac is then probed through the stent mesh with a SL10-MC and closed with a total of 6 platinum micro-spirals. (**C**) Two-years angiographic control shows complete aneurysm occlusion (**D**).

All procedures were performed on a biplane angiosuite (Philips, Best, the Netherlands or Siemens, Erlangen, Germany) with the patient under general anaesthesia. A bolus of heparin (5000 IU) was administered after placement of the femoral sheath, followed by 1000 IU/h until the end of the procedure. Digital subtraction angiography (DSA) and three-dimensional reconstruction images were used to evaluate the specific aneurysm characteristics and to plan the procedure. In majority of the WEB cases, the device was delivered through a dedicated VIA microcatheter (Sequent Medical, Aliso Viejo, CA, USA) into the aneurysmal sac (Fig. 2). Following WEB device types were used: WEB Dual-Layer [DL], WEB Single-Layer [SL] and WEB Single-Layer Sphere [SLS]. The use of additional devices was left to the neurointerventionalist’s discretion.

**Figure 3:**
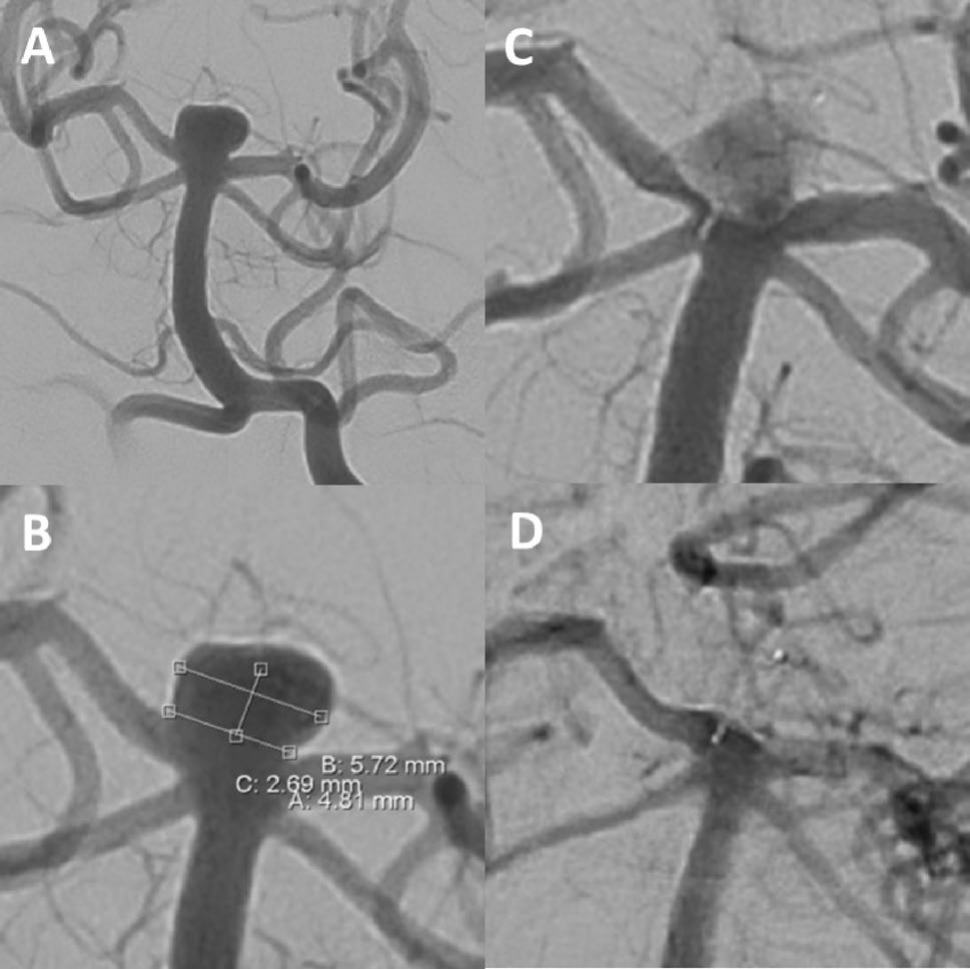
Unruptured aneurysm at the basilar tip (**A + B**). Due to the broad-based geometry and the unruptured aneurysm status, intrasaccular flow-disruption was envisaged. A WEB SL (7 × 3 mm) was placed within the aneurysm sac, achieving immediate contrast stasis (**C**). Two-years angiographic control shows complete aneurysm occlusion (**D**).

In coiling procedures, balloon assistance or stent assistance with Y-, T- or single stenting techniques were used if required at the discretion of the treating neurointerventionalist (Fig. 3). Implanted stent types were: Neuroform EZ and Atlas (Stryker, Alamazoo, MI, USA), Solitaire AB (Covidien, Irvine, CA, USA), eCLIPs (endovascular CLIP System, Evasc, Medical Systems Corp., Vancouver, BC, Canada), LVIS (Micronvention/Terumo, Aliso Viejo, CA, USA), Barrel stent (Barrel Vascular Remodeling Device (VRD) system; Medtronic, Minneapolis, Minnesota, USA), Acclino stent (Acandis, Pforzheim, Germany), LEO Baby (Balt, Duesseldorf, Germany) and Enterprise (Cordis Neurovascular, Miami, FL, USA).

### Anti-aggregation therapy

Specific anti-aggregation therapy regimen was managed in each center as follows:

- *WEB* Acetylsalicylic acid (ASA) 100 mg/day 5–7 days prior the procedure and ASA monotherapy continuation for a minimum of 4 weeks.
- *Stand-alone coiling* No oral anti-aggregation therapy.
- *SAC* Daily dual anti-platelet regimen with ASA 100 mg and clopidogrel 75 mg 5–7 days before treatment and continuation for at least 4 months after the procedure. Thereafter, ASA 100 mg/day monotherapy life-long. In case of non-responsiveness to either drug, dose escalation or substitution with prasugrel (60 mg bolus, 10 mg/day) was applied to counteract.

In cases of unplanned stent-implantations, tirofiban (Aggrastat, Merck, West Point, PY, USA) was administered directly before stent placement and continued for 16–24 h after the procedure, followed by a loading dose of ASA (500 mg) and clopidogrel (300 mg).

### Data collection

The following parameters were retrospectively collected: patient age, sex, aneurysm characteristics (aneurysm dome width, aneurysm height, neck width, dome-to-neck ratio), radiation exposure, procedural-related complications, clinical and angiographic outcome. Procedural radiation exposure was measured as dose area product (DAP [mGy cm^2^]).

### Complications and clinical outcome

All procedure-related complications were recorded. Neurological complications were defined as events with new neurological deficits occurring until last follow-up. Clinical outcome was evaluated by the modified Rankin Scale (mRS) at least at baseline, at discharge and at 6-month follow-up. An increase of the mRS after the procedure was defined as procedural morbidity. A mRS score of > 2 points was considered as unfavourable outcome.

### Angiographic follow-up

Follow-up was generally scheduled at 6 months and 24 months by DSA.

Aneurysm occlusion at follow-up was evaluated using the Raymond-Roy occlusion classification (RROC): 1, complete occlusion, 2, neck remnant, and 3, aneurysm remnant. Complete occlusion and neck remnants were subsumed as adequate occlusion.

### Statistical analysis

Continuous data are presented as means and standard deviation for continuous variables. Normal distribution was tested using the Shapiro–Wilk test. Groups were compared with the unpaired t-test for normally distributed parameters and the Mann–Whitney U test if the data were not normally distributed. Categorical variables are reported as numbers and percentages and groups were compared with the Chi-Square test for cell counts ≥ 5 and the Fisher exact test, if at least one cell count was less than 5. Statistical analysis was performed using SPSS software (IBM SPSS Statistics for Windows, Version 25.0, Armonk, NY, USA). A p-value of < 0.05 was considered as statistically significant.

## Results

### Patient and aneurysm characteristics

The final study population consisted of 40 patients treated by WEB (61.9 years ± 12.6 years) and 35 treated by coiling (63.2 years ± 10.4 years). Baseline patient and aneurysm characteristics are presented in Table 1. The aneurysmal neck width was significantly larger in the WEB group (4.7 ± 1.4 mm) as compared to the coiling group (3.9 ± 1.7 mm; p = 0.028), resulting in a lower D/N ratio in the WEB group. There was no significant difference in the remaining baseline characteristics.

**Table 1:** Baseline patient demographics and aneurysm characteristics.

Parameter	Coiling (N = 35)	WEB (N = 40)	p
Patient age (years)	63.2 ± 10.4	61.9 ± 12.6	0.630
Female sex	29 (83%)	31 (78%)	0.563
Aneurysm dome width (mm)	6.2 ± 1.6	6.2 ± 3.3	0.999
Aneurysm height (mm)	6.3 ± 2.2	5.5 ± 3.2	0.800
Neck width (mm)	3.9 ± 1.7	4.7 ± 1.4	0.028
D/N ratio	1.7 ± 0.7	1.4 ± 0.5	0.035

*D/N ratio* dome/neck ratio.

### Aneurysm treatment and procedural specifics

Procedural specifics are summarized in Table 2. Additional stent implantation was significantly more often necessary for coiled aneurysms (71%, 25/35) than for WEB embolization (2.5%, 1/40, p < 0.001). Eight patients (23%, 8/35) were treated by stand-alone coiling. There was no treatment failure. The treatment duration was shorter in the WEB group (111 ± 57 min) than in the coiling group (152 ± 81 min, p = 0.048). Patient radiation exposure and contrast dye application were lower in the WEB group than in the coiling group, however, without reaching statistical significance (p = 0.088 and p = 0.071, respectively).

**Table 2:** Aneurysm treatment and procedural specifics.

Parameter	Coiling (N = 35)	WEB (N = 40)	p
Coiling/WEB alone, n (%)	8 (23%)	39 (98%)	
Stent-assistance, n (%)	25 (71%)	1 (3%)	
Balloon-assistance, n (%)	2 (6%)	0 (0%)	
**WEB type**			
WEB DL	–	2 (5%)	
WEB SL	–	35 (88%)	
WEB SLS	–	3 (8%)	
WEB 17		15 (38%)	
Treatment duration (min)	152 ± 81	111 ± 57	0.048
Patient radiation exposure (DAP, Gy cm^2^)	107.45 ± 91.11	76.30 ± 62.93	0.088
Contrast dye (ml)	169 ± 72	134 ± 85	0.071

*DL* double layer, *SL* single layer, *SLS* single layer sphere, *DAP* dose area product.

### Complications

A detailed list of complications is presented in Table 3. In the WEB group, two procedure-related thromboembolic events occurred (5.0%). In the first patient, multiple posterior cortical infarcts occurred, probably due to thromboembolism. The patient showed numbness in the left lower leg, which resolved within a few weeks. There was no permanent morbidity (mRS 3 before and after intervention).

**Table 3:** Complications.

Parameter	Coiling (N = 35)	WEB (N = 40)	p
Overall complications	4 (11%)	2 (5%)	0.409
Neurological complications	2 (6%)	1 (2.5%)	0.467
Permanent sequelae	1 (3%)	0	0.467
Thromboembolic event	4 (11%)	2 (5%)	0.409
Cerebral infarction	2 (6%)	1 (2.5%)	0.596
Hemorrhagic event	0	0	1.0
Procedural morbidity	3 (9%)	1 (2.5%)	0.334
Unfavourable outcome	0	0	1.0
Unfavourable outcome at FU	1 (3%)	0	0.467
Mortality	0	0	1.0

*FU* follow-up.

In the second patient, a separation thrombus during WEB implantation could be resolved with intraarterial administration of tirofiban. The patient had no neurological deficits (mRS 0).

In the coiling group, thromboembolic complications occurred in 4 patients (11%). Thereof, 2 events were associated with neurological deficits due to cerebral infarctions. In the first patient, infarction occurred after accidental occlusion of the posterior cerebral artery resulting in homonymous hemianopsia. In the second patient, an acute stent occlusion occurred after 4 months after discontinuation of clopidogrel. The patient presented with dysarthria, vertigo, nausea and vomiting (mRS 4). In the remaining two patients with asymptomatic thromboembolic events, a separation thrombus could be successfully resolved with tirofiban. Moreover, an asymptomatic stent dislocation) occurred in one patient after T-stenting with proximal deployment of the dislocated stent in the left vertebral V3/4 segment.

There were no symptomatic haemorrhagic events and no mortality. The morbidity rates were 9% in the coiling group and 2.5% in the WEB group (p = 0.334). There was no mortality. Overall complication rates and complications subtypes were not statistically different between the groups.

### Angiographic outcome

Angiographic results are summarized in Table 4. Initial angiographic control showed complete or near-complete occlusion in 55% after WEB implantation and in 94% after coiling (p < 0.001).

**Table 4:** Angiographic outcome.

Parameter	Coiling	WEB	p
**Immediate occlusion**	N = 35	N = 40	
RROC = 1, n (%)	33 (94%)	16 (40%)	< 0.001
RROC = 2, n (%)	0 (0%)	6 (15%)	
RROC = 3, n (%)	2 (6%)	18 (45%)	
**Mid-term FU**	N = 29	N = 28	
FU duration (months)	5 ± 2.4	3.7 ± 3.2	0.085
RROC = 1, n (%)	29 (100%)	25 (89%)	0.194
RROC = 2, n (%)	0 (0%)	1 (4%)	
RROC = 3, n (%)	0 (0%)	2 (7%)	
Stable occlusion*, n (%)	28 (97%)	18 (64%)	
Progressive occlusion*, n (%)	1 (3%)	10 (36%)	
Recurrence*, n (%)	0 (0%)	0 (0%)	
**Long-term FU (months)**	N = 20	N = 18	
FU duration	23.0 ± 12.2	20.0 ± 13.8	0.497
RROC = 1, n (%)	17 (85%)	17 (94%)	0.387
RROC = 2, n (%)	1 (5%)	1 (6%)	
RROC = 3, n (%)	2 (10%)	0 (0%)	
Stable occlusison**, n (%)	17 (85%)	13 (72%)	
Progressive occlusion**, n (%)	0 (0%)	5 (28%)	
Recurrence**, n (%)	2 (10%)	0 (0%)	
Retreatment, n (%)	2 (10%)	1 (3%)	1.0

*RROC* Raymond–Roy Occlusion Classification, *FU* follow-up.

*vs. Immediate postinterventional occlusion.

**vs. Mid-term occlusion.

At mid-term follow-up (WEB: 3.7 ± 3.2 months, coiling: 5 ± 2.4 months), complete or near-complete aneurysm occlusion was obtained 100% in the coiling group and in 92.9% in the WEB group (p = 0.194).

At long-term follow-up (WEB: 20.0 ± 13.8 months, coiling: 23.0 ± 12.2 months), complete or near complete occlusions were obtained in 85% after coiling and in 100% after WEB implantation (p = 0.387).

During the follow-up period, there were two aneurysm remnants in the coil group which required retreatment (10%). One patient in the WEB group (3%) was subjected to retreatment with stent-assisted coiling 5 months following the initial treatment, because of a primary non obliterated small recess between aneurysmal wall and WEB. Retreatment rates were not significantly different between the groups (p = 1.0).

## Discussion

In the current study, treatment of unruptured BAT aneurysms by WEB or coiling was associated with similar complication rates, morbidity and angiographic outcome. However, stent-assistance was significantly more often necessary for coiling than for the WEB. Moreover, procedural time, radiation exposure and contrast dye application were lower in the WEB group.

In order to increase the comparability of the study groups, we included only aneurysms that were considered to be amenable to both treatment modalities by an experienced consultant neurointerventionalist, which is a strength of the current study.

### Procedure

BAT aneurysms can have a complex anatomy such as a wide aneurysm-neck, an unfavourable dome-to-neck ratio and the incorporation of vessel origins into the aneurysm base. These aneurysms are typically difficult or technically impossible to treat by stand-alone coiling and require therefore advanced neurovascular techniques such as balloon- or stent-assistance^5,7,21^. With numerous different stent types and stenting techniques (such as Y- or X-stenting) being available, the majority of BAT has become amenable to stent-assisted coiling. Moreover, SAC allows a higher coil packing density, which usually translates into lower recurrence rates. Limitations of stent-implantation include higher complication rates, in particular thromboembolic events and a necessity of long-term anti-platelet therapy^9,22,23^.

In contrast, the WEB device is a self-expandable, barrel-like device which is implanted completely inside the aneurysm sac leaving the parent artery unaffected. The WEB has been mostly used for wide-necked bifurcation aneurysms, as its barrel-like shape provides good anchorage in the majority of these aneurysms^15,24–27^. Given a careful size selection, originating vessel from the aneurysm base could be spared with the WEB.

Adjunctive stent implantation for the WEB may become necessary, if the lower part of the device tends to protrude into the parent vessel or when the WEB narrows the parent artery. In the literature, around 8% of reported WEB procedures required stent support^28^.

In the current study, stent implantation rates were significantly higher in the coiling group than in the WEB group (71% vs. 3%). This result favours the WEB group, as stent implantation is usually associated with higher thromboembolic complication requires and the necessity for long-term anti-platelet therapy. The use of additional stents also translated into a higher procedure time and a higher radiation exposure compared to the coiling group. From a cost-efficiency-perspective, this aspect also attenuates the differences in treatment costs, which are per se high in the WEB group due to relatively high material costs.

During the study period, WEB was increasingly used for wide-necked saccular-shaped aneurysms as alternative treatment option for SAC. In our opinion, aneurysms with this morphology and a diameter < 11 mm are well suited for the WEB. Larger and morphologically more complex aneurysms are not optimal candidates for the WEB and should be treated by SAC, flow-diversion, or clipping or by a combination of these modalities.

### Efficiency

Previous studies comparing the WEB with coiling or stent-assisted coiling demonstrated comparable mid-term angiographic results for the three groups^20,29^. For instance, a systematic metanalysis by Lv et al. comprising 18 studies reported mid-term adequate occlusion rates of 81%^30^. In our study, mid-term adequate occlusion rates were slightly higher (92.9%). A limitation of these studies was that long-term angiographic outcome has not been systematically analysed. While coiling is generally associated with relatively high recanalization rates within a long-term follow-up period^7,31–33^, recent studies indicate that WEB occlusion is relatively stable beyond a one-year follow-up period^34^.

As the WEB provides progressive thrombosis of the aneurysm sac via the flow-disrupting effect, the initial angiographic results favoured the WEB over the coiling group (complete occlusion 94% vs. 40%). However, at mid-term and long-term follow-up, the complete and near-complete occlusion rates as well as the retreatment rates were comparable between both groups, indicating similar efficiency.

While stent-assisted coiling of BAT aneurysms is associated with sufficiently high angiographic results, previous studies indicate high recanalization rates for conventional coiling of BAT aneurysms. For instance, Henkes et al. also reported complete or near-complete occlusion after conventional coiling of BAT aneurysms in 86% directly after the procedure, 70% after 1 year, 56% after 2 years and 48% at longer follow-up^5^. Although these results indicate a high recanalization rate, a direct comparison with our data is difficult, as the cited studies included also very large and giant aneurysms, while the present study focused on aneurysms ≤ 11 mm. There is broad evidence that aneurysm size correlates positively with aneurysm recurrence.

### Complications

The most frequent complications related to WEB treatment are thromboembolic complications, mainly due to the formation of a separation thrombus during the procedure^35^. Delayed complications are rarely reported for the WEB. Likewise, we observed no delayed complications in the WEB group in 445 cumulative patient-months. In comparative studies, WEB and coiling had similar thromboembolic event rates, while stent-assisted coiling may be associated with increased ischemic stroke and morbidity rates.

In the current series focusing on BAT aneurysms, in the coiling group, thromboembolic complications occurred in 11% which is in the range of large coiling series on BTAs that are reported between 6 and 12%^5,7,21,33,36^. In the WEB group, thromboembolic complications occurred in 5%. Likewise, thromboembolic complication rates were 9.4% in the study by Arthur et al.^26^ and 14.4% in the study by Pierot et al.^16^. Consequently, the thromboembolic event rate of the coiling group was twice as high than that in the WEB group, however, without reaching statistical significance. The lack of significance may rely on the small study sample and warrants further investigation by a larger study. Of note, all symptomatic thromboembolic events in the coiling group occurred in the subgroup of aneurysms treated with stent-assistance, which is in line with the results of previous studies.

Aneurysms treated with the WEB had a significantly wider neck than coiled aneurysms in the current series. In this context, Fiorella et al. identified a wide aneurysm neck as an independent risk factor for procedural complication rates^37^. In summary, these results indicate at least equivalent safety of WEB and (stent-assisted) coiling.

### Limitations

The major limitations of this study are its non-randomized retrospective design, moderate sample size and the self-assessment of the aneurysm occlusion grades. Moreover, long-term follow-up was not available for all patients, which can bias long-term clinical and angiographic results. Finally, the choice of treatment method was based on the discretion of the neurointerventionalist. Thus, we cannot exclude any potential bias towards one particular technique or adjunctive device.

## Conclusions

The results of this comparative study indicate a similar safety and efficacy profile of (stent-assisted) coiling and WEB embolization of unruptured intracranial aneurysms for a selected subset of aneurysms that are amenable to either treatment modality. However, stent-assistance was significantly less frequently required for the WEB than for coiling. This result may be considered as an advantage for the WEB, as stent-assistance requires long-term anti-platelet medication. In contrast, the WEB does not require postinterventional anti-platelets as a purely intrasaccular device. Ultimately, the choice of the treatment modality should be made based on the specific aneurysm characteristics, the individual experience of the neurointerventionalist and patient preference.

## Supplementary Information

Supplementary Information.

## Data Availability

All data generated or analysed during this study are included in this published article [and its Supplementary Information files].
